# Non-inferiority of 1 month *versus* longer dual antiplatelet therapy in patients undergoing PCI with drug-eluting stents: a systematic review and meta-analysis of randomized clinical trials

**DOI:** 10.1177/20406223221093758

**Published:** 2022-05-17

**Authors:** Gani Bajraktari, Ibadete Bytyçi, Artan Bajraktari, Michael Y. Henein

**Affiliations:** Institute of Public Health and Clinical Medicine, Umeå University, 901 87 Umeå, Sweden; Clinic of Cardiology, University Clinical Centre of Kosova, University of Prishtina, Prishtina, Kosovo; Institute of Public Health and Clinical Medicine, Umeå University, Umeå, Sweden; Clinic of Cardiology, University Clinical Centre of Kosova, University of Prishtina, Prishtina, Kosovo; Institute of Public Health and Clinical Medicine, Umeå University, Umeå, Sweden; Clinic of Cardiology, University Clinical Centre of Kosova, University of Prishtina, Prishtina, Kosovo; Institute of Public Health and Clinical Medicine, Umeå University, Umeå, Sweden

**Keywords:** acute coronary syndrome, dual antiplatelet therapy, percutaneous coronary intervention, stable coronary artery disease

## Abstract

**Aim:**

The aim of this meta-analysis was to evaluate the safety of 1-month dual antiplatelet therapy (DAPT) followed by aspirin or a P2Y12 receptor inhibitor, after percutaneous coronary intervention (PCI) with drug-eluting stents (DES), based on the available evidence.

**Methods:**

PubMed, MEDLINE, Embase, Scopus, Google Scholar, CENTRAL, and ClinicalTrials.gov database search identified four RCTs of 26,431 patients who underwent PCI with DES and compared 1-month *versus* >1-month DAPT. The primary endpoint was major bleeding and co-primary endpoint stent thrombosis, and secondary endpoints included all-cause mortality, cardiovascular death, myocardial infarction (MI), stroke, and major adverse clinical events (MACE).

**Results:**

Compared with >1-month DAPT, the 1-month DAPT was associated with a similar rate of major bleeding (OR = 0.74, 95%CI: 0.51–1.07, *p* = 0.11, *I*^2^ = 67%), stent thrombosis (OR = 1.10, 95%CI: 0.82–1.47, *p* = 0.53, *I*^2^ = 0.0%), similar risk for all-cause mortality (OR = 0.89, 95%CI: 0.77–1.04, *p* = 0.14, *I*^2^ = 0%), CV death (OR = 0.80, 95% CI: 0.55–1.60, *p* = 0.24, *I*^2^ = 0.0%), MI (OR = 1.02, 95% CI: 0.88–1.19, *p* = 0.78, *I*^2^ = 0.0%), and stroke (OR = 0.76, 95% CI: 0.54–1.08, *p* = 0.13, *I*^2^ = 29%). The risk of MACE was lower (OR = 0.84, 95% CI: 0.73–0.98, *p* = 0.02, *I*^2^ = 39%) in the 1-month DAPT compared with the >1-month DAPT. Only patients with stable CAD had lower risk of MACE with 1-month DAPT (OR = 0.81, 95% CI: 0.67–0.98, *p* = 0.03, *I*^2 =^ 21%) compared with >1-month DAPT.

**Conclusion:**

This meta-analysis proved the non-inferiority of 1-month DAPT followed by aspirin or a P2Y12 receptor inhibitor compared with long-term DAPT in patients undergoing PCI with DES.

## Introduction

Coronary artery disease (CAD) is a major cause of morbidity and mortality worldwide, with a cumulative total mortality rate of 18% at 5 years in patients with acute coronary syndrome (ACS).^1,2^ In such patients, percutaneous coronary intervention (PCI) is the treatment of choice, particularly in those with stable CAD (sCAD). Following PCI and implantation of drug-eluting stent (DES), the current guidance recommends 6–12 months of dual antiplatelet therapy (DAPT), with aspirin and P2Y12 receptor inhibitors, as a protective measure against arterial thrombosis.^3,4^ However, it has been shown that DAPT increases the risk of bleeding, which offsets the expected benefits of reduced ischemic events;^4–6^ hence, attempts to shorten the duration of DAPT to a maximum of 1 month have been sought and evaluated.^7,8^ These studies showed non-inferiority of 1-month DAPT followed by aspirin or a P2Y12 receptor inhibitor compared with conventional long-term treatment with DAPT. Such promising findings have not reached clinical guidelines yet. Recently, two more randomized clinical trials showed similar results in patients with high bleeding risk^9^ and in all comers^10^ undergoing PCI. The objective of the present systematic review and meta-analysis was to provide a comprehensive evaluation of the short-term (1 month) DAPT in patients undergoing PCI with DES by performing a meta-analysis including the most recent randomized controlled trials (RCTs).

## Methods

We followed the PRISMA guidelines of the 2020 preferred reporting items for systematic reviews and meta-analysis statement.^11^ Due to the study design (meta-analysis), neither Institutional Review Board (IRB) approval nor patient informed consent was needed.

### Search strategy

We systematically searched PubMed-Medline, EMBASE, Scopus, Google Scholar, the Cochrane Central Registry of Controlled Trials, and ClinicalTrial.gov, up to September 2021, using the following key words: (‘percutaneous coronary intervention’ OR ‘PCI’) AND (‘drug-eluting stent’) AND ( dual antiplatelet therapy’ OR ‘DAPT’) AND (‘randomized controlled trial’ OR ‘RCT’) AND (‘1-month OR ‘one-month’). In this meta-analysis, we also included abstracts from selected congresses: Scientific Sessions of the American Heart Association (AHA), European Society of Cardiology (ESC), American College of Cardiology (ACC), and European Society of Atherosclerosis (EAS). Only articles published in English were included in the analysis. No filters were applied. G.B. and I.B, independently and separately evaluated all articles. The finally selected articles were obtained in full text and were searched carefully by the same two researchers, who extracted necessary data and evaluated the quality of articles. Disagreements were resolved by discussion with a third party (M.Y.H.).

### Eligibility criteria

Studies eligible for inclusion were those fulfilling the following criteria: (1) randomized design comparing the efficacy and safety of 1-month DAPT with that of more than 1-month DAPT treatment in patients who underwent PCI for ACS or stable CAD; (2) minimum follow-up period of 10 months; and (3) full-text studies published in peer-reviewed journals. *Exclusion criteria were*: (1) non-randomized studies; (2) unpublished papers; and (3) ongoing trials. Observational and unpublished studies were not included in the meta-analysis.

### Data extraction

Qualified studies were searched and the following data were collected, including: (1) first author’s name; (2) date of publication; (3) clinical trial name; (4) place where the study was conducted; (5) number of centers involved; (6) study design; (7) number of patients in each of the two study arms who received 1-month or >1-month DAPT treatment; (8) follow-up period; and (9) detailed clinical outcome and nature of events in the two groups.

### Outcomes and definitions

#### Endpoints

The primary endpoint was major bleeding based on Bleeding Academic Research Consortium (BARC) type 3 or 5 bleeding, and major or clinically relevant non-major bleeding was defined as a bleeding event of BARC type 2, 3, or 5.^12^ The co-primary endpoint was stent thrombosis according to the Academic Research Consortium (ARC) definitions.^13^ Secondary endpoints included all-cause death, cardiovascular (CV) death, major adverse clinical events (MACE), myocardial infarction (MI), and stroke. MACE was defined as the combination of all-cause death, CV death, MI, major bleeding, stent thrombosis, and stroke.

Acute MI was defined according to symptoms, electrocardiographic signs, and biomarker elevation above the upper normal limit.^13,14^ Stroke was defined as an acute symptomatic episode of neurological dysfunction more than 24 h in duration in the absence of therapeutic intervention and neuroimaging evidence of cerebral, spinal, or retinal tissue injury.^15^

#### Quality assessment

Risk-of-bias assessment in the included studies was evaluated by the same investigators for each study and was performed systematically using the revised Cochrane RoB2 tool involving five domains (randomization process, deviation from intended interventions, missing outcome data, outcome measurement, and selection of reported results).^16^ The risk of bias in each study was conventionally classified as ‘low’, ‘high’, or ‘unclear’ (Supplemental Table S1).

#### Statistical analysis

We performed the pooled analyses of treatment effects and clinical outcomes using the Cochrane Collaborative software, RevMan 5.3.5 (the Nordic Cochrane Center, the Cochrane Collaboration, 2014, Copenhagen).^17^ Baseline characteristics are reported as median and range. Mean and standard deviation (SD) values were estimated using the method described by Hozo *et al*.^18^ Analysis is presented in forest plots. A two-tailed *p value* <0.05 was considered significant. Meta-analyses were performed using the fixed-effects model, and the random-effects model was used if heterogeneity was encountered. Heterogeneity between studies was assessed using Cochrane *Q* test and *I*^2^ index, with *I*^2^ <25% indicating low, 25–50% moderate and >50% high heterogeneity.^19^ Based on hazard ratio value of 1, above or below, we calculated the relative risk for CV events.^20^ Publication bias was assessed using Egger’s test and visual inspection of funnel plots. To test the possible effect of trials with large sample size on direction of clinical outcomes, we performed influence analysis.

## Results

### Search results and trial flow

Of the 3420 articles identified in the initial searches, 30 studies were initially considered as potentially relevant. After a stringent selection process, four articles met the inclusion criteria^7–10^ (Supplemental Figure S1).

### Characteristics of the included studies

Four studies, with a total of 26,431 patients, met all the inclusion criteria, 13,191 patients had been randomized to receive DAPT for 1 month, and 13,240 received DAPT for more than 1 month according to conventional recommendations (Supplemental Table S2). The mean follow-up duration was 14.8 months (Table 1) in the two groups.

**Table 1.** Main characteristics of trials included in the study.

Study (trial) year	Study design	Center location	Population	Sample size (1 M/>1 M)	Primary endpoints	Secondary endpoints	Follow-up
GLOBAL LEADERS 2018	RCTs (double-blinded)	130 centers (18 countries)	ACS sCAD	15,968 (7980/7988)	Major bleeding Stent thrombosis	All-cause death Stroke, MI, MACE	24 months
STOPDAPT-2 2019	RCTs (double-blinded)	Japan	ACS sCAD	3009 (1500/1509)	Major bleeding Stent thrombosis	All-cause death CV death, MI Stroke, MACE	12 months
MASTER DAPT 2021	RCTs, (double-blinded)	140 centers (30 countries)	ACS sCAD PCI	4434 (2204/2230)	Major bleeding Stent thrombosis	All-cause death CV death, MI Stroke, MACE	11.2 months
ONE MONTH DAPT 2021	RCTs, (double-blinded)	23 centers (South Korea)	ACS sCAD	3020 1507/1513)	Major bleeding Stent thrombosis	All-cause death CV death, MI Stroke, MACE	12 months

ACS, acute coronary syndrome; CV, cardiovascular; MACE, major adverse cardiac events; MI, myocardial infarction. sCAD, stable coronary artery disease;

### Demographic and clinical data of 1-month DAPT *versus* >1-month DAPT

The two patient groups were not different in age (68.8 ± 10.2 *versus* 69.1 ± 9.1 years, *p* = 0.77) and the proportion of female participants (41.6% *versus* 40.5%, *p* = 0.53, respectively). Similarly, cardiac risk factors of the 1-month DAPT group were almost similar to those of the >1-month DAPT: arterial hypertension (72.8% *versus* 72.9%; *p* = 0.89), diabetes (34.1% *versus* 33.8%; *p* = 0.43), dyslipidemia (72.9% *versus* 73.7%; *p* = 0.67), and current smokers (19.8% *versus* 17.8%; *p* = 0.11). Prior coronary intervention procedures and events including coronary artery bypass grafting (CABG), PCI, MI, and bleeding were also not different between the two treatment groups (*p* > 0.05 for all; Table S3).

### Angiographic data of 1-month DAPT *versus* >1-month DAPT

Angiographic data of the 1-month DAPT group were not different from the >1-month DAPT patients: left main (LM) disease (2.9% *versus* 2.53%; *p* = 0.13), left anterior descending artery (LAD) (46.5% *versus* 41.6%; *p* = 0.10), left circumflex artery (LCx) (21.4% *versus* 22.9%; *p* = 0.50), right coronary artery (RCA) (30.4% *versus* 28.9%; *p* = 0.33), and CABG (7.1% *versus* 6.5%; *p* = 0.22). Patients in the two treatment groups, 1 month and >1 month, did not differ in the number of vessels treated per patient: 1, 2, or 3 (*p* > 0.05 for all). Moreover, access site procedures were not significantly different between the two treatment groups (*p* > 0.05 for all; Table S4)

### Primary outcomes

**Figure 1.**
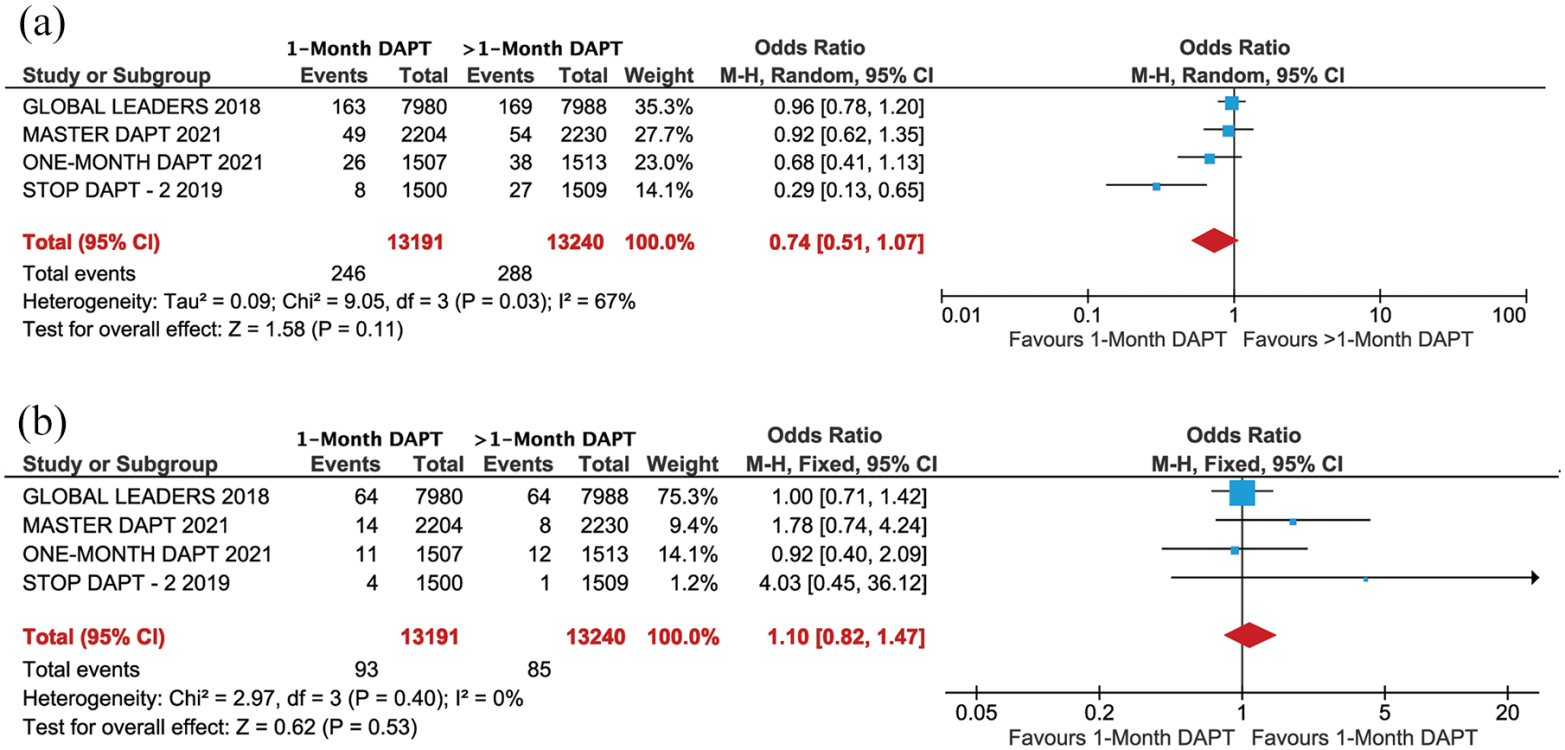
Forest plot comparing primary outcome between 1-month DAPT and >1-month DAPT: (a) major bleeding and (b) stent thrombosis.

The treatment group of 1-month DAPT had similar risk for major bleeding [1.86% *versus* 2.17%; odds ratio (OR) = 0.74, 95% confidence interval (CI): 0.51–1.07, *p* = 0.11, *I*^2^ = 67%] and stent thrombosis (0.70% *versus* 0.64%; OR = 1.10, 95% CI: 0.82–1.47, *p* = 0.53, *I*^2^ = 0.0%) to the group who received >1-month DAPT (Figure 1(a) and (b)).

### Secondary outcomes

**Figure 2.**
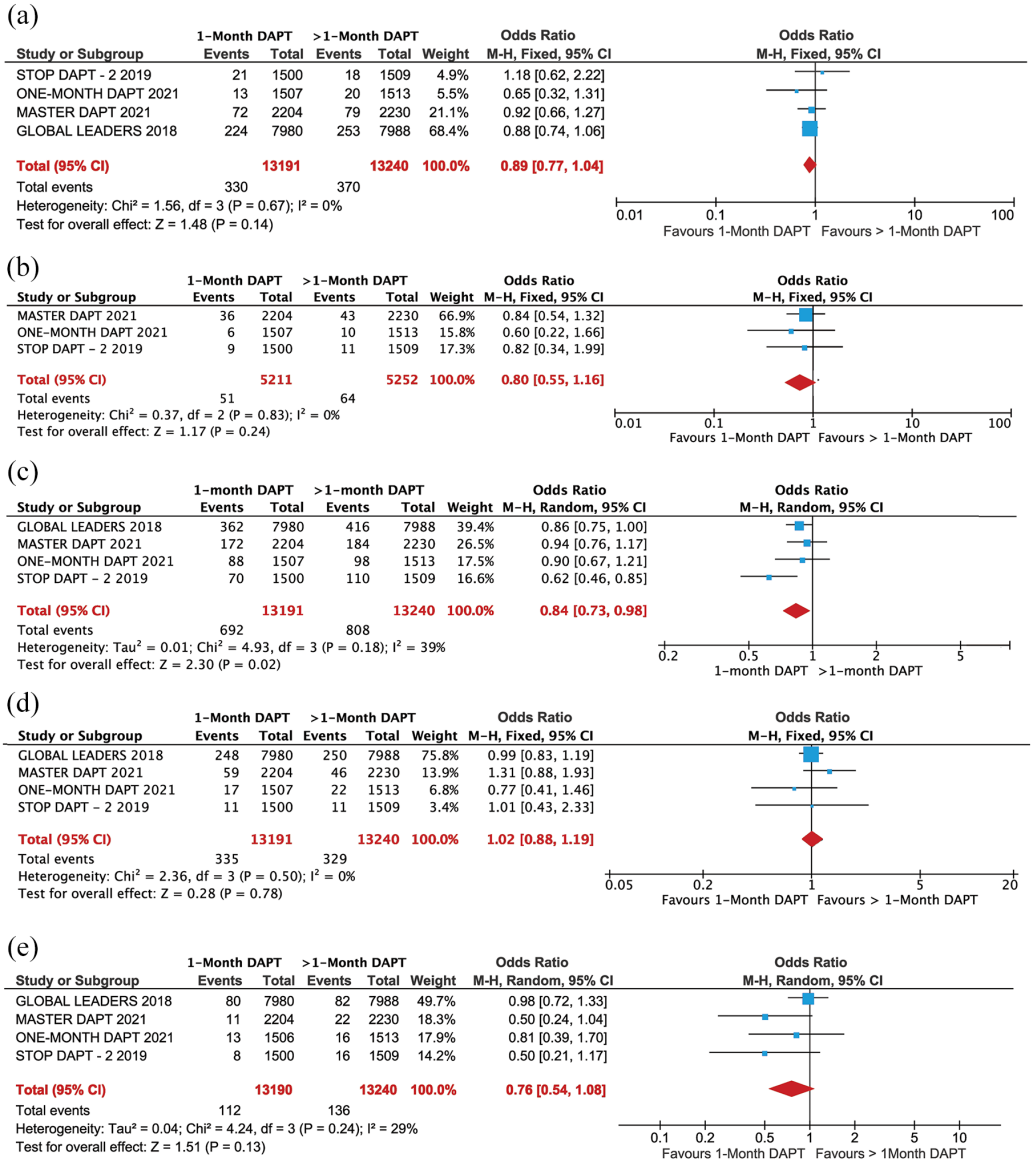
Forest plot comparing secondary outcome between 1-month DAPT and >1-month DAPT: (a) all-cause, (b) CV death, (c) MACE, (d) MI, and (e) stroke.

**Figure 3.**
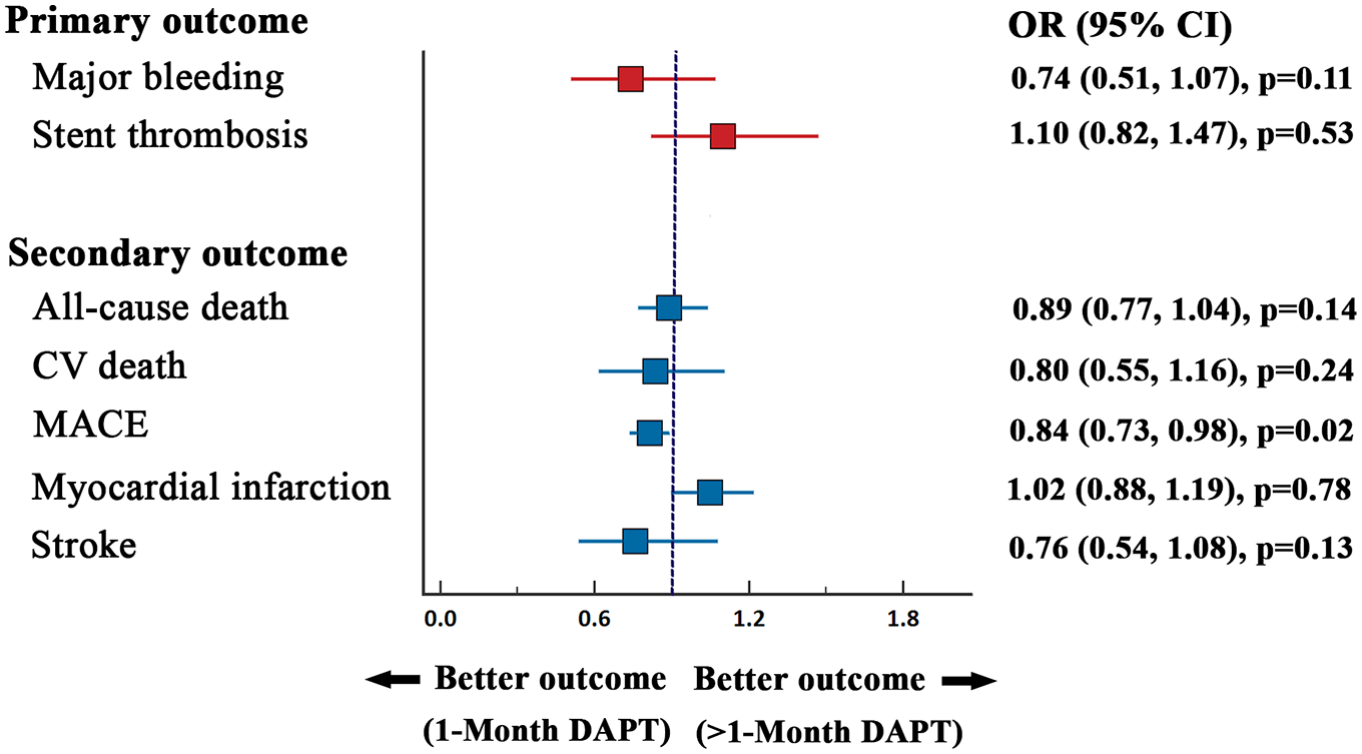
Summary forest plot comparing primary and secondary outcome between 1-month DAPT and >1-month DAPT.

The two treatment groups had similar risk for all-cause mortality (2.50% *versus* 2.79%; OR = 0.89, 95% CI: 0.77–1.04, *p* = 0.14, *I*^2^ = 0.0%), CV death (0.97% *versus* 1.21%; OR = 0.80, 95% CI: 0.55–1.16, *p* = 0.24, *I*^2^ = 0.0%), MI (2.52% *versus* 2.48%; OR = 1.02, 95% CI: 0.88–1.19, *p* = 0.78, *I*^2^ = 0.0%), and stroke (0.85% *versus* 1.02%; OR = 0.76, 95% CI: 0.54–1.08, *p* = 0.13, *I*^2^ = 29%; Figure 2(a), (b), (d), and (e)). In contrast, the risk of MACE was lower (5.2% *versus* 6.10%; OR = 0.84, 95% CI: 0.73–0.98, *p* = 0.02, *I*^2^ = 39%; Figure 2(c)) in patients who received 1-month DAPT compared with those who received >1-month DAPT. The summary of outcomes is presented in Figure 3.

**Figure 4.**
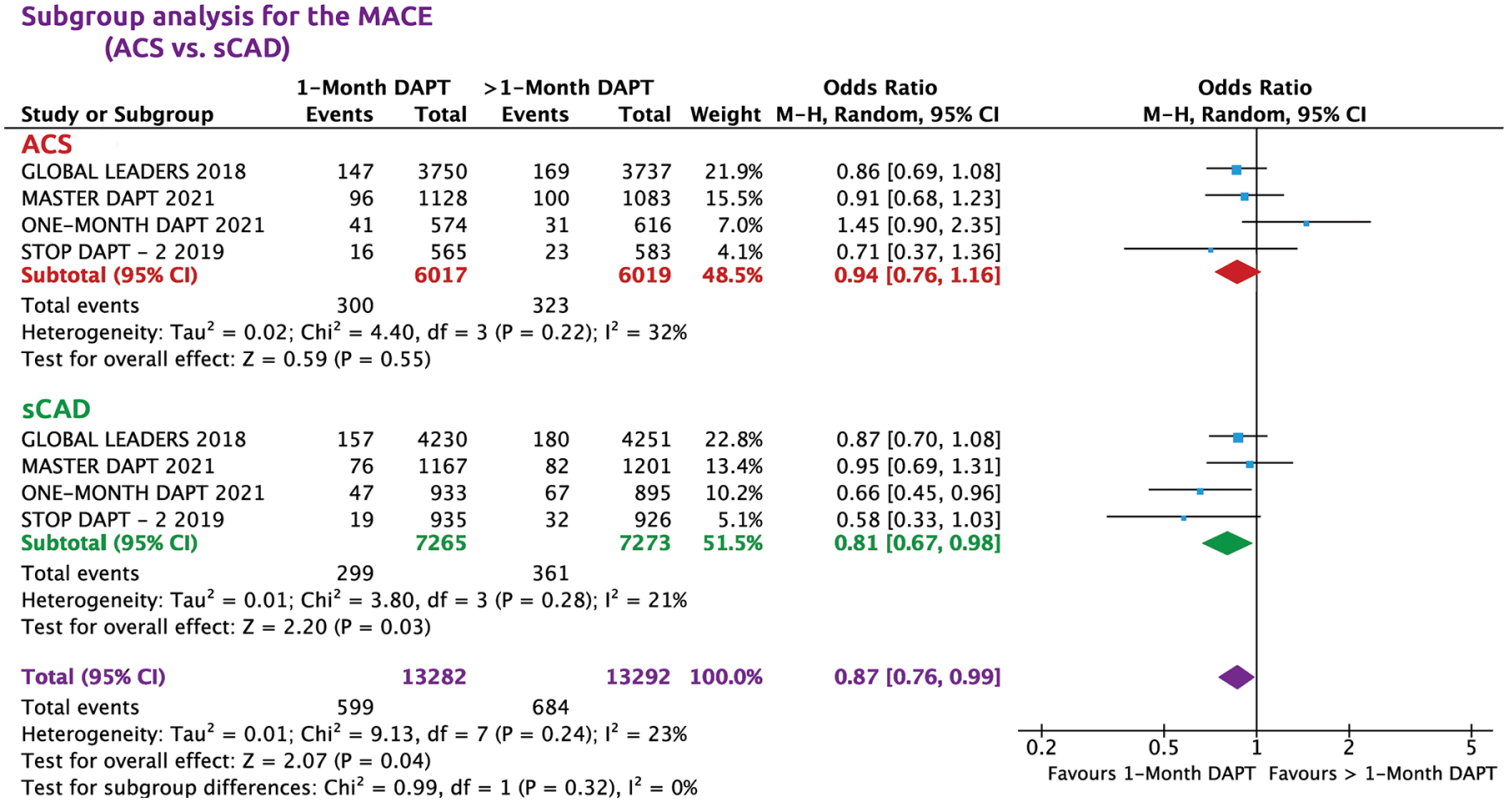
Subgroup analysis for MACE (ACS *versus* sCAD).

Subgroup analysis for MACE has shown that in patients with ACS, the risk for MACE was similar in the two treatment groups (OR = 0.94, 95% CI: 0.76–1.16, *p* = 52, *I*^2^ = 32%), while in stable CAD the risk of MACE was lower in the 1-month DAPT (OR = 0.81, 95% CI: 0.67–0.98, *p* = 0.03, *I*^2^ = 21%) compared with >1-month DAPT (Figure 4).

#### Influence analysis

The influence analysis was used to test the trials with large sample size (⩾2000 *versus* < 2000 patients per arm). One-month DAPT was associated with lower risk of MACE in large trials (*p* = 0.04), while in small trials it was not significant due to the influence of 1-month DAPT trial (Supplemental Figure S2). A sub-analysis of 1-month DAPT showed lower MACE in the stable CAD but higher in ACS patients receiving 1-month DAPT compared with >1-month DAPT (Figure 4).

## Discussion

The main findings of this meta-analysis can be summarized as follows: (1) the 1-month DAPT followed by aspirin or a P2Y12 receptor inhibitor was not inferior in protecting patients against stent-related thrombosis, compared with long-term DAPT; (2) 1-month DAPT was not inferior for all-cause mortality, cardiac death, MI, or stroke, compared with longer DAPT; (3) 1-month DAPT had similar risk for major bleeding compared with >1-month DAPT; and (4) it was also associated with less MACE (combination of all-cause mortality, CV death, major bleeding, stent thrombosis, and stroke) compared with >1-month DAPT.

### Data interpretation

Atherosclerosis in general and as a cause for CAD, in particular, is an important health issue, being the main contributor for the existing high rate of morbidity and mortality worldwide,^21^ despite the significant recent advances in medical and interventional treatments. PCI is the commonest intervention used for coronary artery stenosis, both stable and ACS.^3,4^ Scientific advances in PCI have demonstrated that the use of DES carries better chance for short- and long-term maintenance of stent patency, compared with the early generations of bare metal stents, even in critical lesions, for example, LM stem disease.^22^ However, stent thrombosis after PCI remains an important complication which has not been optimally reduced/avoided by DES, even with the new generation.^23^ In view of this ongoing clinical problem, current clinical guidelines recommend 6–12 months treatment with DAPT (aspirin and a P2Y12 receptor inhibitors) as a protective measure against in-stent thrombosis, in all patients undergoing PCI for ACS or significant stable CA stenosis.^3,4^ However, despite the documented clinical benefit of DAPT, they carry important risk for bleeding, particularly in patients at high bleeding risk.^4–6^ Therefore, researchers have envisaged balancing the risk of stent thrombosis with that of bleeding by attempting the use of 1-month DAPT treatment and assess its potential benefit/risk ratio. Few RCTs were approved to compare 1-month DAPT with standard longer use of DAPT as recommended by current guidelines.

Patients included in this meta-analysis were from the four currently available RCTs,^7–10^ who were classified into two groups, 1 month and >1 month of DAPT, which they received after PCI with DES. Our analysis showed that 1-month DAPT was not inferior to 6–12 months of treatment in protecting patients from in-stent thrombosis, irrespective of the age, gender, cardiac risk factors, and prior coronary intervention procedures and events (CABG, PCI, MI, and bleeding). In fact, 1-month DAPT was associated with less MACE compared with > 1-month treatment, probably as a result of the cumulative effect of bleeding, death, and stroke, which were lower in patients with 1-month treatment. The similar mortality between groups we found, whether overall or CV, suggests a potential safe place for 1-month DAPT treatment in interventional CAD management irrespective of the nature of the disease, stable or unstable.

Stents are considered foreign body to the human arteries; hence, thrombosis becomes an inevitable potential complication, which could occur within hours, in some cases. Our findings have shown that such risk could be safely overcome within 1-month DAPT, suggesting a process of fast endothelialization. The less MACE in the same group of 1-month DAPT compared with >1-month DAPT is likely related to the multiple risk factors these patients carry, despite the lack of difference between the two groups, thus suggesting a multifactorial impact.

### Clinical implications

This study provides a strong evidence supporting the safety of using 1-month DAPT compared with the conventionally recommended 6–12 months treatment following PCI for CAD. The thrombosis rate was not different between the two approaches as were the other conventional demographics and cardiovascular risk factors. These findings suggest non-inferiority of the 1-month DAPT approach compared with >1 month.

### Clinical limitations

The number of available RCTs that complied with our inclusion criteria was small, but the analysis findings were consistently uniform through them. We did not have any hand in the data collection, but we trust the integrity of the trials published and their data, whose results were not significantly dissimilar. The lack of heterogeneity between studies was also additional supportive evidence for the high standard data collection, analysis, and consistent conclusions. Although some of the data we analyzed were previously published, our detailed analysis adds extra scientific weight supporting the use of 1-month DAPT treatment after PCI and DES, irrespective of the type of angina.

## Conclusion

This meta-analysis proved non-inferiority of 1-month DAPT followed by aspirin or a P2Y12 receptor inhibitor compared with long-term DAPT, in patients undergoing PCI with DES.

## Supplemental Material

sj-doc-1-taj-10.1177_20406223221093758 – Supplemental material for Non-inferiority of 1 month versus longer dual antiplatelet therapy in patients undergoing PCI with drug-eluting stents: a systematic review and meta-analysis of randomized clinical trials Click here for additional data file. Supplemental material, sj-doc-1-taj-10.1177_20406223221093758 for Non-inferiority of 1 month versus longer dual antiplatelet therapy in patients undergoing PCI with drug-eluting stents: a systematic review and meta-analysis of randomized clinical trials by Gani Bajraktari, Ibadete Bytyçi, Artan Bajraktari and Michael Y. Henein in Therapeutic Advances in Chronic Disease
