# Supplementary material for: Non-inferiority of 1 month versus longer dual antiplatelet therapy in patients undergoing PCI with drug-eluting stents: a systematic review and meta-analysis of randomized clinical trials
Source: Ther Adv Chronic Dis. 2022 May 17;13:20406223221093758. doi: 10.1177/20406223221093758 (PMC9118452; doi:10.1177/20406223221093758)
Supplement: sj-doc-1-taj-10.1177_20406223221093758 – Supplemental material for Non-inferiority of 1 month versus longer dual antiplatelet therapy in patients undergoing PCI with drug-eluting stents: a systematic review and meta-analysis of randomized clinical trials [file sj-doc-1-taj-10.1177_20406223221093758.doc]

**SUPPLEMENTARY DATA:**

**
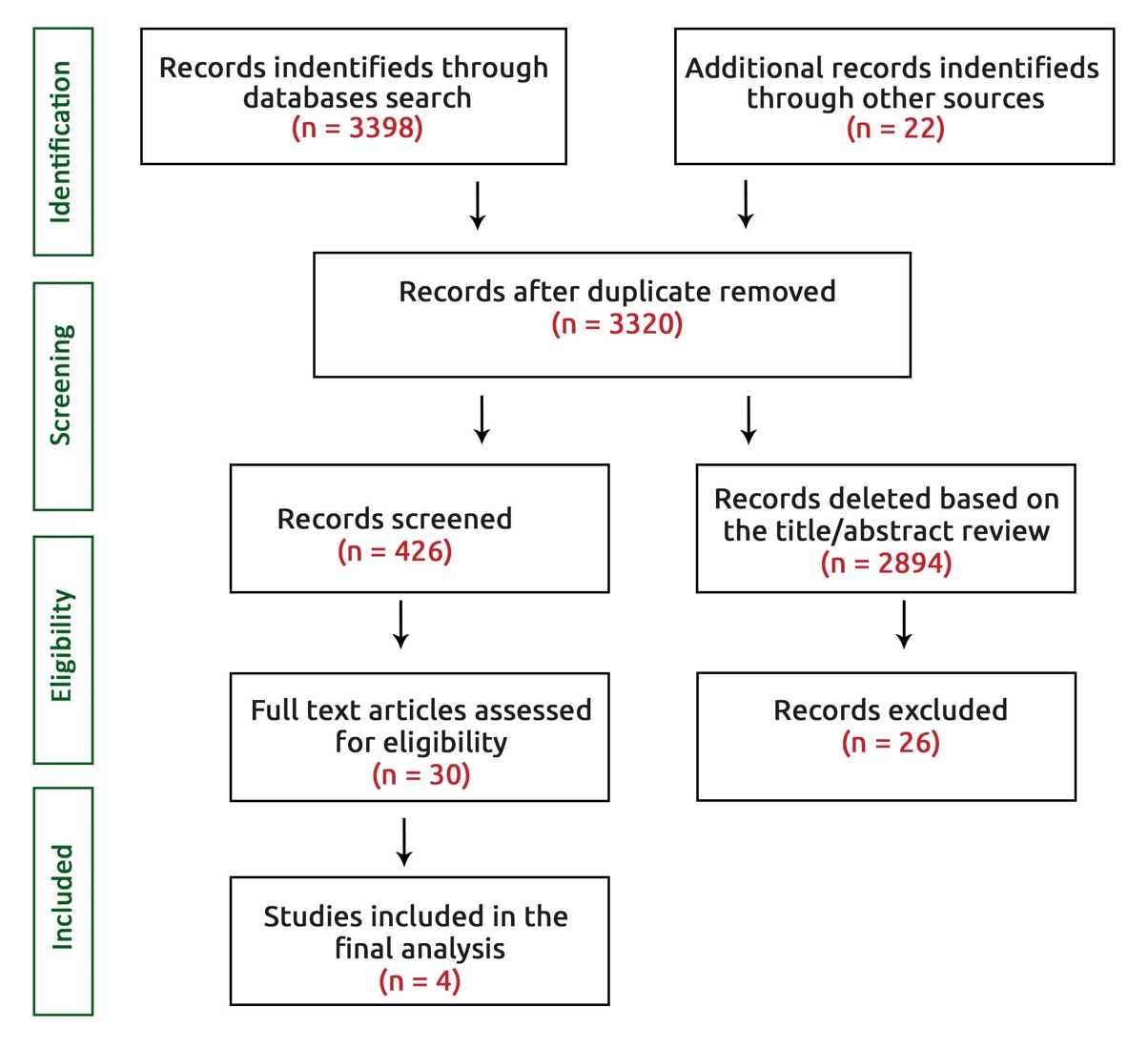
**

**FigureS1.** Flow chart of trials

**Table S1.** Assessment of risk of bias in the included studies using RoB2 for RCTs studies.

| **Study, year** | **Randomization** | **Deviation** | **Missing** | **Measurement** | **Selection** | **Overall** |
| --- | --- | --- | --- | --- | --- | --- |
|  | **process** | **from intended** | **outcome** | **of the** | **of the reported** |  |
|  |  | **interventions** | **data** | **outcome** | **results** |  |
| GLOBAL LEADERS 2018 | L | L | L | L | L | L |
| STOPDAPT-2 2019 | L | L | L | L | L | L |
| MASTER DAPT 2021 | L | L | L | L | L | L |
| ONE MONTH DAPT 2021 | L | L | L | L | L | L |

L: Low; S: Some concerns; H: High

| ***Overall risk-of-bias judgement*** | ***Criteria*** |
| --- | --- |
| *Low risk of bias* | *The study is judged to be at low risk of bias for all domains for this result.* |
| *Some concerns* | *The study is judged to raise some concerns in at least one domain for this result, but not to be at high risk of bias for any domain.* |
| *High risk of bias* | *The study is judged to be at high risk of bias in at least one domain for this result. The study is judged to have some concerns for multiple domains in a way that substantially lowers confidence in the result.* |

**Table S2. Antiplatelet regimen treatment in patients enrolled among trials included in the study**

| **Study (trial) year** | **1-Month DAPT regimen** | **>1-Month DAPT regimen** |
| --- | --- | --- |
| GLOBAL LEADERS 2018 | 75–100 mg aspirin daily in combination with 90 mg ticagrelor twice daily for 1 month, followed by 90 mg ticagrelor twice daily for 23 months | 12 months of dual antiplatelet therapy consisting of 75–100 mg aspirin daily in combination with either 75 mg clopidogrel daily (for patients with stable coronary artery disease) or 90 mg ticagrelor twice daily (for patients with acute coronary syndromes) |
| STOPDAPT-2 2019 | 1 month of DAPT followed by clopidogrel monotherapy | 12 months of DAPT with aspirin and clopidogrel |
| MASTER DAPT 2021 | 1 month of DAPT followed by single antiplatelet therapy consisted of aspirin or a P2Y12 inhibitor | Continued dual antiplatelet therapy for at least 6 months after the index procedure, or for those receiving clinically indicated oral anticoagulation, for at least 3 months after the index procedure |
| ONE MONTH DAPT 2021 | 1 month of DAPT followed by aspirin monotherapy | 6-12 months of DAPT followed by aspirin monotherapy |

**Table S3. Main characteristics of patients enrolled among trials included in the study**

| **Study (trial) year** | **Groups** | **Age** | **Female** | **HTN** | **DM** | **Dyslipidemia** | **Smoking** |  | | **Prior** | | |
| --- | --- | --- | --- | --- | --- | --- | --- | --- | --- | --- | --- | --- |
|  |  | **year** | **%** | **%** | **%** | **%** | **%** | **MI** | **PCI** | | **CABG** | **Bleeding** |
| GLOBAL LEADERS 2018 | 1-Month DAPT | 64.5±10 | 76.6 | 74 | 25.7 | 69.3 | 25.9 | 23 | 32.7 | | 5.6 | 0.6 |
|  | >1-Month DAPT | 64.6±10 | 76.9 | 73.3 | 24.9 | 70 | 26.3 | 23.6 | 32.7 | | 6.2 | 0.7 |
| STOPDAPT-2 2019 | 1-Month DAPT | 68±10 | 21.1 | 73.7 | 39 | 74.4 | 26.6 | 13.8 | 33.5 | | 1.1 | 1.3 |
|  | >1-Month DAPT | 69±10 | 23.4 | 74 | 38 | 74.8 | 20.6 | 13.2 | 35.1 | | 2.8 | 1.9 |
| MASTER DAPT 2021 | 1-Month DAPT | 76 ±8.7 | 38 | 76.9 | 32.9 | 67 | 10 | 18.9 | 25.9 | | 7.4 | 7.2 |
|  | >1-Month DAPT | 76 ± 8.8 | 30.7 | 78.2 | 34.3 | 68 | 8.1 | 18.8 | 26 | | 7.5 | 6.8 |
| ONE MONTH DAPT 2021 | 1-Month DAPT | 67 ±10 | 31 | 67 | 37 | 81 | 17 | 4 | 16 | | 1 | NR |
|  | >1-Month DAPT | 67 ±10 | 31 | 66 | 38 | 82 | 16 | 4 | 18 | | 2 | NR |

Abbreviations: HTN: hypertension; DM: diabetes mellitus; MI: myocardial infarction; PCI: percutaneous coronary intervention; CABG: coronary artery by-pass grafting; NR: non-reported.

**Table S4. Angiographic characteristics of patients enrolled among trials included in the study**

| **Study (trial) year** | **Groups** | **Treated vessel per patients** | | | | | **No of vessels treated** | | | **Access site** | | | | |
| --- | --- | --- | --- | --- | --- | --- | --- | --- | --- | --- | --- | --- | --- | --- |
|  |  | **LM** | **LAD** | **LCx** | **RCA** | **CABG** | **One** | **Two** | **Three** |  | **R** | **F** | **B** | **FFR** |
| GLOBAL LEADERS 2018 | 1-Month DAPT | 1.9 | 42 | 24.3 | 31.6 | 1.1 | 74.6 | 20.5 | 5.0 |  | 73.9 | 26.3 | 0.6 | NR |
|  | >1-Month DAPT | 1.1 | 23.4 | 23.5 | 30.7 | 1.0 | 74.7 | 19.8 | 5.5 |  | 74.2 | 26.1 | 0.6 | NR |
| STOPDAPT-2 2019 | 1-Month DAPT | 2.9 | 55.2 | 17.9 | 29.1 | 0.2 | NR | NR | NR |  | 82.1 | 13.5 | NR | 14.2 |
|  | >1-Month DAPT | 2.5 | 56.6 | 22.2 | 27.2 | 0.2 | NR | NR | NR |  | 83.3 | 11.9 | NR | 13.4 |
| MASTER DAPT 2021 | 1-Month DAPT | 3.9 | 42.3 | 22.1 | 30.5 | 19.9 | 74.8 | 21 | 4.2 |  | 84.1 | 15.8 | 0.2 | 7.2 |
|  | >1-Month DAPT | 4.0 | 42.9 | 23 | 28.8 | 18.1 | 72.2 | 23.7 | 4.1 |  | 86.9 | 12.8 | 0.3 | 6.8 |
| ONE MONTH DAPT 2021 | 1-Month DAPT | NR | NR | NR | NR | NR | 43 | 33 | 25 |  | NR | 20 | NR | NR |
|  | >1-Month DAPT | NR | NR | NR | NR | NR | 42 | 31 | 27 |  | NR | 20 | NR | NR |

Abbreviations: LM: Left main; LAD: Left atrial descending artery; LCx: Left circumflex; RCA: Right coronary artery; CABG: Coronary artery bypass graft; R: Radial; F: Femoral; B: Brachial; FFR: Fractional flow reserve; NR: Non-reported;

**Figure S2. Influence analysis of effect of sample size on MACE; a) MACE in large trial; b) MACE in small trial)**

**
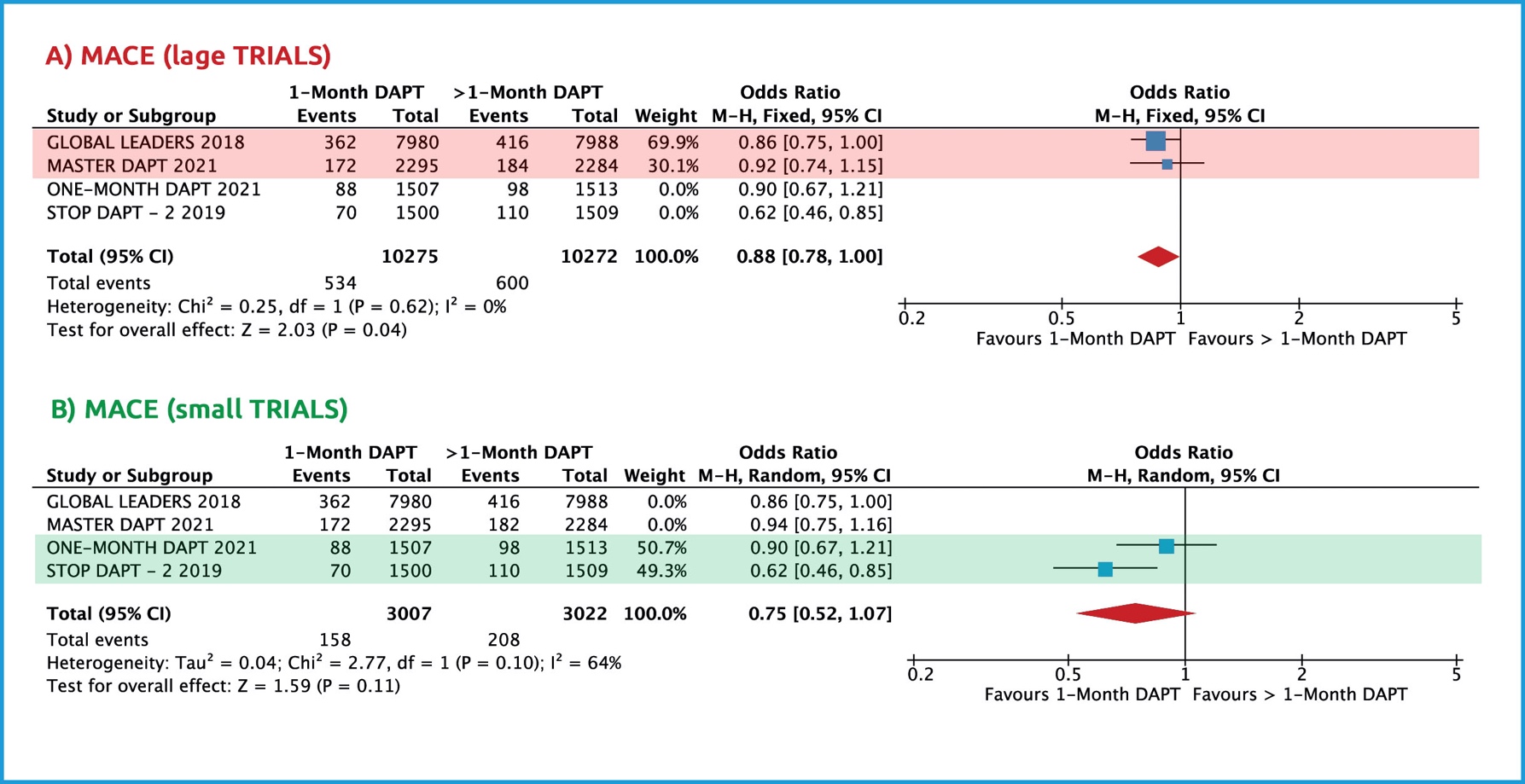
**
